# Practical Study of Malaria

**Published:** 1910-02

**Authors:** 


### BOOKS AND AUTHORS.

**Practical Study of Malaria.** By **William H. Deaderick**, M.D., Member American Society of Tropical Medicine; Fellow London Society of Tropical Medicine and Hygiene. Octavo of 400 pages, with 100 illustrations, some in colors. Philadelphia and London: W. B. Saunders Co., 1909. (Cloth, $4.50 net.)

This book was written by a physician who practises medicine in Arkansas. If he treats malaria as well as he writes on the subject, his patients are to be congratulated. The work is a strong and practical presentation of the subject on which it treats. Its literary style and composition, are also of the best.

The author states in his preface that his wife did much of the copying and proof reading, and he generously accords her a share of credit for the production of his book. We hope to hear from this combination again. Any physician who reads the treatise, and everyone should read it, will conclude that the Deaderick firm is a strong one. J. A. R.

